# Persistence of colicinogenic *Escherichia coli *in the mouse gastrointestinal tract

**DOI:** 10.1186/1471-2180-9-165

**Published:** 2009-08-12

**Authors:** Osnat Gillor, Itamar Giladi, Margaret A Riley

**Affiliations:** 1 Zuckerberg Institute for Water Research, J Blaustein Institutes for Desert Research, Ben-Gurion University, Sde Boker Campus 84990, Israel; 2 Department of Life Sciences, Ben-Gurion University, Beer-Sheva 84105, Israel; 3 Department of Biology, University of Massachusetts Amherst, Amherst, MA 01003, USA

## Abstract

**Background:**

The ability of a bacterial strain to competitively exclude or displace other strains can be attributed to the production of narrow spectrum antimicrobials, the bacteriocins. In an attempt to evaluate the importance of bacteriocin production for *Escherichia coli *strain residence in the gastrointestinal tract, a murine model experimental evolution study was undertaken.

**Results:**

Six colicin-producing, yet otherwise isogenic, *E. coli *strains were administered and established in the large intestine of streptomycin-treated mice. The strains' persistence, population density, and doubling time were monitored over a period of 112 days. Early in the experiment only minor differences in population density between the various colicin-producing and the non-producing control strains were detected. However, over time, the density of the control strains plummeted, while that of the colicin-producing strains remained significantly higher (F_(7,66) _= 2.317; P < 0.0008).

**Conclusion:**

The data presented here support prior claims that bacteriocin production may play a significant role in the colonization of *E. coli *in the gastrointestinal tract. Further, this study suggests that the ability to produce bacteriocins may prove to be a critical factor in determining the success of establishing probiotic *E. coli *in the gastrointestinal tract of humans and animals.

## Background

The gastrointestinal (GI) tract of humans is colonized by *Escherichia coli *within about 40 hours of birth [1]. This facultative anaerobe is then stably maintained as a relatively minor, but critical, component of the large intestine microflora with a cell density approximately 1000 times lower than the predominant bacterial genera, such as *Bacteriodes*, *Clostridia*, and anaerobic streptococci. *E. coli *adheres to, and primarily subsists on, the mucin layer that coats the epithelial cells of the large intestine. A dominant, resident strain will normally persist in the GI tract for periods of months to years, until it is eventually replaced by one of the many transient strains continually passing through the intestinal lumen. The basis for these periodic shifts is not known and has recently become the focus of a large body of research [2].

In part, this increased interest in the dynamics of *E. coli *strains is due to dysbiosis, or microbial imbalances of the normal human microflora of the GI tract. This common outcome of antibiotic therapies is now considered to be a contributing factor to many chronic and degenerative diseases such as irritable bowel syndrome and rheumatoid arthritis [2]. Attempts to re-establish a healthy microbial flora, alleviate GI disorders, and control pathogenic *E. coli *in humans and animals through the administration of probiotics, are limited by our ability to choose and properly administer the most appropriate strains [3,4]. Understanding strain dynamics of *E. coli *in the GI tract may provide a more sound approach to both probiotic strain choice and methods of administration [5-8].

One powerful predictor of the ability of a strain of *E. coli *to competitively exclude or displace other strains is the production of one or more of a large family of narrow spectrum antimicrobials, the bacteriocins. Theoretical studies have shown that bacteriocin production enhances the invasion and establishment success of the producing strains [9,10]. *In vivo *studies further demonstrate that bacteriocin production improves the establishment success of its producing strain [11]. Similar results were obtained when mice harboring bacteriocin-sensitive strains were co-caged with mice harboring bacteriocin-producing strains. Within a relatively short period (three to five weeks) the sensitive strains had been displaced by the bacteriocin-producing strains [12].

*E. coli *are prolific producers of their own species-specific bacteriocins, known as colicins, which were first identified over 80 years ago [13], and given the name colicin to identify the producing species. The frequency of colicin production varies among *E. coli *populations depending on the host species diet [14], the relatedness of the *E. coli *strains present [15], and the habitat quality [16]. However, on average, forty percent of the strains in any population are likely to produce one or more colicins [17,18].

Over thirty colicins have been characterized to date, all of which are plasmid-encoded, high molecular weight proteins that are induced in times of stress [19]. Upon release of colicins from the producing cell, the toxins kill their targets primarily by membrane permeabilization or nucleic acid degradation [20]. Genes encoding colicin functions are found in clusters that include a toxin-encoding gene; an immunity gene, encoding a protein conferring self-specific protection to the cell against its own colicin; and, frequently, a lysis gene, encoding a protein involved in colicin release via lysis or pseudo-lysis of the producing cell [19].

It has recently been suggested that bacteriocin production is a critical factor in determining the establishment success of probiotic bacteria in humans and animals [21]. To investigate this hypothesis, we introduced *E. coli *strains differing only in the carriage and identity of bacteriocin-encoding plasmids into the GI tract of mice. The importance of bacteriocin production in colonization and persistence of their *E. coli *hosts in the mouse intestine was elucidated over time providing a rare and novel glimpse into the impact of bacteriocins on the establishment of enteric bacteria in the mouse GI tract.

## Results

This study was designed to examine the colonization and persistence of colicinogenic *E. coli *strains in the mouse GI tract following a single administration. To this end, six isogenic strains of *E. coli *BZB1011 were created differing in only two characters: (i) the ability to produce a colicin (determined by the presence or absence of a plasmid encoding a colicin gene cluster); and (ii) the identity of the colicin produced (one of the following colicins: A, E1, E2, E7, K, and N). Mice treated with streptomycin to eradicate their resident enterobacterial flora were inoculated with streptomycin resistant bacteriocin producing (or non producing control) strains that were then monitored for 112 days by weekly sampling of mouse pellets.

### The persistence and population density of colicin producers in the mouse GI tract

**Figure 1:**
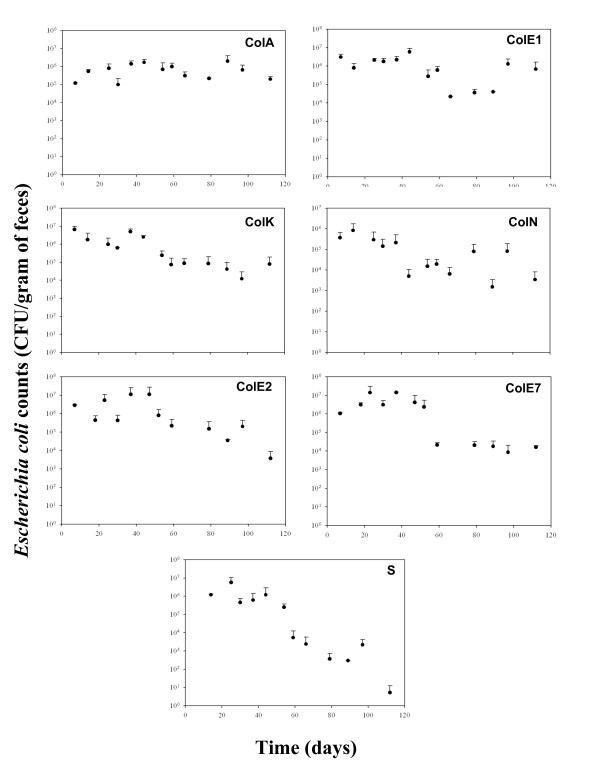
**Colonization of the mouse intestine by colicin producing *E. coli *strains**. Each point represents the mean CFU (g feces)^-1 ^determined for two mice in each of two cages. Bars represent the standard error of the log10 for each point. The number of cells measured at day 112 for the colicin free strain falls below the limit of detection determined at 10^2 ^CFU (g feces)^-1^.

Figure 1 reports the average number of bacterial colony forming units (CFUs) detected over the course of the experiment, with each point representing an average taken over four mice (two cages with two mice per cage) per colicin treatment. A separate graph is provided for each of the seven colicin treatments employed. Subsamples of isolated colonies were used to verify the strain's colicin phenotype by examining their ability to (i) grow in the presence of their own colicin extract; and (ii) produce a clearing zone in a lawn prepared from a colicin sensitive strain (data not shown). Four patterns of strain dynamics emerged: First, one week after each mouse was inoculated, all of the strains had successfully established in the mouse GI tract at relatively high densities, with an average of 10^5^-10^7 ^CFUs (g feces)^-1^. Second, two colicin treatments (A and E1) showed no difference in the average number of CFUs measured over the course of the experiment, with an average of 7.5 × 10^5 ^and 1.4 × 10^6 ^CFUs (g feces)^-1^, respectively. Third, four of the colicin treatments (E2, E7, K and N) showed a steady, slow decline in density over the course of the experiment, with average initial and final densities of 2.4 × 10^6 ^and 2.6 × 10^4 ^CFUs (g feces)^-1^, respectively. Fourth, relative to all other treatments, the non-colicin producing control strain declined most rapidly and was undetectable in samples from day 112 (< 10^2 ^CFU (g feces)^-1^).

A statistically significant difference in strain persistence was observed over the course of the experiment (time × strain, Repeated Measure Analysis, F_(7,66) _= 2.317, P < 0.0008). A second repeated-measure ANOVA, which excluded the colicin-free control strain, revealed significant difference in persistence times among the colicin strains (time × strain, Repeated Measure ANOVA, F_(6,55) _= 1.896, P < 0.009). These results indicate that the significant differences in strain abundance observed over time is not solely due to the rapid disappearance of the colicin free control strain and presumably reflect differences between the more slowly declining strains (colicins K, N, E2 and E7) and the highly persistent strains (colicins A and E1).

One week after the initial strain introduction into the mouse GI tract, no significant differences in density were observed between the different colicin-producing strains (one-way ANOVA at t = 0, F_(6,7) _= 0.136, P = 0.98; no significant contrasts). A simple one-way ANOVA indicated no such differences at the end of the experiment either (one-way ANOVA at t = 112 days, F_(6,5) _= 3.28, P = 0.1). However, the orthogonal contrasts analysis indicated a significant difference in the density of the control strain versus all other colicinogenic strains (t_(5) _= 3.63, P = 0.015).

### The doubling time of colicin producers isolated from the mouse GI tract

An average strain generation time was determined from five colonies isolated from each colicin treatment at days 0 and 112 (Table 1). An increase in doubling time was observed for all strains, ranging from 6–33% relative to day 0 (two-way ANOVA, F_(1,48) _= 84.42, P < 0.001). However, the degree of increase varied among strains, as indicated by a significant interaction term (time × strain, two-way ANOVA, F_(6,48) _= 3.26, P = 0.006), with the non-colicin producing strain experiencing the greatest increase in generation time (Table 1).

**Table 1:** Growth rate of *E. coli *strains over time

*Mode of Action*	*E. coli *strains		Growth rate μ^1^	
			*0 days*	*112 days*

Pore formation	BZB1011 pColA-CA31	(ColA)	0.56 ± 0.03	0.51 ± 0.02
	
	BZB1011 pColE1-K53	(ColE1)	0.54 ± 0.03	0.51 ± 0.04
	
	BZB1011 pColK-K235 (ColK)		0.54 ± 0.03	0.47 ± 0.05
	
	BZB1011 pColN-284	(ColN)	0.57 ± 0.02	0.48 ± 0.02

DNA degradation	BZB1011 pColE2-P9	(ColE2)	0.57 ± 0.01	0.45 ± 0.04
	
	BZB1011 pColE7-K317	(ColE7)	0.53 ± 0.02	0.42 ± 0.05
	
	BZB1011	(S)	0.61 ± 0.03	0.41 ± 0.07

^1^Growth rate is expressed in generations/h.

## Discussion

The abundance and diversity of bacteriocin production in microbial populations point to the fundamental role these potent toxins serve in mediating strain dynamics in microbial systems. Indeed, most species of bacteria have been shown to possess bacteriocins [20] and levels of production within a species can be as high as 95%. For example, nearly 40% of the *E. coli *isolated from fecal samples of animals and humans were shown to be colicinogenic [17,18], while greater than 95% of the *Pseudomonas aeruginosa *isolated from environmental and clinical sources are bacteriocin producers [22].

Numerous *in silico *and *in vitro *studies have shown that colicinogenic *E. coli *rapidly out-compete their colicin sensitive counterparts, due to the lethality of colicin production [9,23,10]. In the present study, the average increase in the generation time of producer strains was lower then that monitored for the colicin free cells (Table 1). Similar to other *E. coli *strains established in streptomycin-treated mice [24], we observed an increase of ~30% in doubling time, while the colicinogenic strains increase in generation time was more moderate (~12% in average). In contrast, in an *in vivo *study of bacteriocins employing the same mouse model as described here, did not detect an increased persistence of colicinogenic enteric bacteria [24]. However, in that study persistence was monitored for only 15 days. Our data suggest that over a longer period of time, 112 days in the present study, the benefit of colicinogenicity becomes more apparent (Figure 1), with colicin producers maintaining significantly higher densities than their non-colicin producing counterparts. The colicin-based advantage observed in the present *in vivo *study reflects a similar advantage to colicin production as has been detected in prior *in silico *and *in vitro *studies [20]. Our results are even more promising with respect to the advantage gained from colicin production when the sampling method employed here is considered, as fecal-based sampling will generally underestimate the actual density of the strain in the GI tract [25,26].

There is one further colicin-based *in vitro *study, which employed the same mouse model described here, but which differed significantly in experimental design. In this latter study the focus was on the interaction (or competition) between colicinogenic and non-colicinogenic strains, while the current study focuses on the ability of colicinogenicity to enhance strain maintenance [12]. This prior colicin competition study revealed that colicin production enhances strain persistence when mice equilibrated with colicin producing strains are co-caged with mice equilibrated with colicin sensitive strains [12]. Thus, although the intent of the two studies is quite different, both reveal that colicinogenicity has a significant and positive effect on the ability of a strain to be maintained in the GI tract of a streptomycin-treated mouse. Many studies in humans and livestock have shown that probiotic bacteria have the ability to re-establish an indigenous microflora after perturbations of the normal intestinal flora [27-31]. Probiotic bacteria provide this health benefit in many ways and the production of toxins, in particular bacteriocins, was proposed as a leading candidate in this process [21]. *E. coli *strain Nissle 1917, a producer of microcins H47 and M [32], is a well characterized probiont in humans and livestock [3,5,33,8]. This strain was found to be effective in treating chronic inflammatory bowel disease [33] and in inhibiting the adhesion of enteric pathogens to the GI epithelial cells of infants [5]. *E. coli *strain H22 inhibits the invasion of the enetric pathogen *Shigella flexneri *in germ-free mice, probably due to the production of microcin C7 [34], colicins E1 and Ib, as well as aerobin and an unidentified phage [4]. In order for a probiotic strain to exert its beneficial effect in the GI tract, it is essential for the cells to become established. Feeding trials with a variety of probiotic strains have shown that the strains often disappear from the GI tract within weeks of administration [25,35]. Several studies have examined methods to increase strain persistence using prebiotics [36]; synbiotic dietary supplements [26]; and addition of uptake systems. This latter mechanism involves inserting the listerial betaine uptake system, BetL [37], into the probiotic strains such as *Bifidobacterium breve *strain UCC2003 [38] and *Lactobacillus salivarius *strain UCC118 [39]. The present study suggests that production of a bacteriocin may serve a similar beneficial function.

## Conclusion

We have shown that bacteriocin-producing strains of *E. coli*, but not their bacteriocin-free counterparts, were recovered from the feces of mice over extended periods of sampling following a single administration of the strains. These results suggest that colicinogenicity is beneficial in increasing *E. coli *persistence in the mouse GI tract.

## Methods

### Bacterial strains

Six bacteriocin-encoding plasmids were chosen for this study because they encode two of the most common killing mechanisms, pore formation and nucleic acid degradation [40], known in enteric produced bacteriocins. Moreover, the selected bacteriocins bind to their targets via a range of cell-surface receptors (e.g., BtuB, OmpF and Tsx) and use various translocation systems (e.g., TolA and B) [19]. Finally, theses bacteriocins are all encoded on small, non conjugative plasmids implying similar cost of carriage to the host [19].

A streptomycin-resistant mutant of *E. coli *strain BZB1011 [12] was chemically transformed [41]. Briefly, cells were grown in Luria Broth (LB; Sigma, St. Louis, MO) overnight, seeded in fresh medium to grow to OD_600 _0.3–0.4. The cells were then washed twice with ice-cold 100 mM of CaCl_2 _(Sigma, St. Louis, MO) and diluted to yield 10^7^-10^8 ^cells in 100 μl aliquots. A total of 2 ng of the bacteriocin's plasmid DNA were added to each aliquot, mixed gently, and placed on ice for 30 min. The tubes were transferred to a water bath at 42°C for exactly 90 s and transferred back to an ice bath for 1–2 min. A total of 100 μl of 10× LB medium were added to each tube and incubated in a 37°C water bath for 60 min. Transformants were spread on LB plates previously coated with the corresponding bacteriocin lysate. The emerging colonies were isolated and their phenotype examined as described below (see phenotypic determination section).

Each of the resulting strains (the six colicin plasmid-bearing strains as well as the colicin-free, isogenic control strain) was established in two pairs of co-caged mice. Fourteen cages (two per strain) were established and the co-caged mice were permitted to interact freely. Cell density and killing phenotypes of the resident *E. coli *strain in each mouse were monitored by fecal pellet plating (see below).

### Growth conditions

Luria broth (LB) and agar (Difco, Lawrence KS), and MacConkey agar (Sigma, St. Louis, MO) were prepared according to manufacturer's instructions. M9 minimal medium was prepared as previously described [41]. Cultures were grown at 37°C with shaking at 200 rpm. Mouse innocula were prepared from LB overnight cultures started from a single colony on LB agar plates. The cultures were pelleted, washed and resuspended in phosphate buffered saline (Sigma, St. Louis, MO) to a final concentration of 10^9 ^bacteria ml^-1^.

### Growth kinetics

Growth kinetics were measured in minimal media (M9) with strains isolated at the beginning (day 0) and end (day 112) of the experiment. Generation time was determined for the inoculated strain (day 0) and for five single colonies isolated from the caged mice (one or two isolates per mouse) at day 112. Overnight cultures grown in M9 media were diluted and grown to early exponential phase (*A*_600 _≈ 0.2) and culture aliquots (25 μl) were inoculated into the wells of sterile, transparent, 96-well microtiter plates. The plates were incubated in an Infinite M200 (Tecan, Grödig, Austria) microplate reader at 37°C with orbital shaking. The optical density was monitored every 20 min at 600 nm wavelength and the generation time of each colony was calculated. Growth kinetics for each strain was measured in triplicate during each of three replicate growth assays.

### Mice inoculation and sampling

The mouse study was performed in compliance with federal guidelines for the ethical treatment of animals with oversight by the Institutional Animal Care and Use Committee. Animals were kept in a conventional animal colony and all experiments were approved by the animal ethics committee of Yale University. A total of 28 mice were treated with streptomycin to eradicate their enterobacterial flora and were then inoculated with the streptomycin resistant BZB1011 control strain or one of the six colicinogenic strains (four mice per treatment) and the strains persistence was monitored for 112 days.

Twenty-eight four week-old female CD-1 mice were obtained from Charles River Laboratories (Wilmington, MA). Prior to bacterial inoculation and throughout the experiment, the mice were given 5 g l^-1 ^streptomycin sulfate (Sigma, St. Louis, MO) in their drinking water to eliminate any resident Gram-negative facultative bacteria. After one week of preliminary streptomycin treatment, the mice were screened for fecal enteric bacteria by plating fecal pellets on MacConkey agar plates. All mice were free of detectable enteric bacteria. Overnight cultures of the *E. coli *strains were harvested by centrifugation, washed with PBS, and resuspended in a one-tenth volume of PBS. Colonization of the *E. coli *strains was established by a single administration whereby each animal received 100 μl of ~10^9 ^cells per-os. Fecal samples were taken by transferring the mice to sterile plastic boxes, and collecting their pellets as soon as they were extracted. The pellets were immediately transferred to sterile, pre-weighed tubes containing phosphate buffered saline supplemented with 25% glycerol, weighed and the pellets net weight was calculated. The samples were homogenized and sub-samples were diluted in phosphate buffered saline for plating on selective media (MacConkey agar) supplemented with 100 μg ml^-1 ^streptomycin sulfate. The lower limit of detection in fecal plate counts was 10^2 ^CFU (g feces)^-1 ^for 100 μl of the diluted solution per plate. The remaining samples were stored at -80°C. Colony forming units (CFUs) were monitored per gram feces.

### Phenotypic determination

Crude colicin lysates were prepared according to the procedure of Suit et al [42] and stored at 4°C until use. Twenty colonies of streptomycin-resistant *E. coli *from fecal pellets obtained from each mouse at four-week intervals were assayed for the production of growth inhibition zones on plates pre-inoculated with a sensitive lawn (*E. coli *strain BZB1011). Confirmation of the identity of the colicin produced was provided by a strain's ability to grow in the presence of its own colicins (100 μl of crude colicin lysate spread onto LB plates), due to the immunity protein it produces. The zones of inhibition of each strain were documented using an imaging and documentation system (Bio-Rad, Hercules, CA).

### Statistical analysis

Each cage was treated as an independent sample and an average of the two co-caged mice was determined. The average number of CFUs per cage was compared at two times, 0 and 112 days, using a one-way ANOVA. In addition, for each of these times we employed two orthogonal contrasts to test for differences in CFUs among groups of strains that were chosen *a priori*. One contrast served to compare the average number of CFUs of the colicin-free strain with that of the other (colicinogenic) strains. The second served to compare the average number of CFUs of the colicinogenic strains. A repeated-measure ANOVA was conducted to test for differences in the persistence of the various strains over time treating strain as a between-subject factor and time as a within-subject factor. The effects of strain type and time (i.e. beginning vs. end of the experiment) on strain doubling time were tested with a two-way ANOVA with both strain and time treated as fixed factors. All statistical analyses were done with the STATISTICA 2007 (StatSoft, Tulsa, OK).

## Authors' contributions

OG and MAR conceived and designed the study. OG carried out the microbial and mouse data analyses. IG performed the statistical analysis. OG, IG, and MAR draft, read, and approved the manuscript.
